# Creative Arts Interventions for Stress Management and Prevention—A Systematic Review

**DOI:** 10.3390/bs8020028

**Published:** 2018-02-22

**Authors:** Lily Martin, Renate Oepen, Katharina Bauer, Alina Nottensteiner, Katja Mergheim, Harald Gruber, Sabine C. Koch

**Affiliations:** 1 Research Institute for Creative Arts Therapies (RIArT), Alanus University of Arts and Social Sciences, Alfter/Bonn, Villestr. 3, 53347 Alfter, Germany; Renate.Oepen@alanus.edu (R.O.); harald.gruber@alanus.edu (H.G.); sabine.koch@alanus.edu (S.C.K.); 2 Department for Therapy Sciences, SRH University Heidelberg, Maria-Probst-Str. 3, 69123 Heidelberg, Germany; katharina.bauer@hotmail.de; 3 Robert-Bosch-Klinik, Auerbachstr. 110, 70376 Stuttgart, Germany; Alina.Nottensteiner@web.de; 4 Schoen-Klinik, Hofgarten 10, 34454 Bad Arolsen, Germany; katja.mergheim@gmx.de

**Keywords:** creative arts therapies, arts interventions, art, music, dance, drama, stress management, prevention, systematic review

## Abstract

Stress is one of the world’s largest health problems, leading to exhaustion, burnout, anxiety, a weak immune system, or even organ damage. In Germany, stress-induced work absenteeism costs about 20 billion Euros per year. Therefore, it is not surprising that the Central Federal Association of the public Health Insurance Funds in Germany ascribes particular importance to stress prevention and stress management as well as health enhancing measures. Building on current integrative and embodied stress theories, Creative Arts Therapies (CATs) or arts interventions are an innovative way to prevent stress and improve stress management. CATs encompass art, music, dance/movement, and drama therapy as their four major modalities. In order to obtain an overview of CATs and arts interventions’ efficacy in the context of stress reduction and management, we conducted a systematic review with a search in the following data bases: Academic Search Complete, ERIC, Medline, Psyndex, PsycINFO and SocINDEX. Studies were included employing the PICOS principle and rated according to their evidence level. We included 37 studies, 73% of which were randomized controlled trials. 81.1% of the included studies reported a significant reduction of stress in the participants due to interventions of one of the four arts modalities.

## 1. Introduction

According to the World Health Organization (WHO), stress is currently the world’s most pronounced health risk [1]. Consequences of stress are constant agitation, exhaustion, burnout, helplessness, fear, and eventually a weak immune system or even organ damage [2]. The inability to cope with stress is a risk factor for various epidemiologically significant illnesses: cardiovascular, muscular or skeletal diseases, depression or anxiety disorders [3]. Russ et al. [4] showed that stress and other psychological health problems have an effect on the mortality risk of otherwise healthy individuals. In Germany, for the last 15 years, health insurance companies have registered a drastic increase in stress-induced absenteeism at work [5]. Interviewing 1200 German participants from various religious, age, and milieu backgrounds, the health insurance fund *Techniker Krankenkasse (TK)* [5] found that 6 out of 10 people report to experience stress privately or at work, with 23% out of those feeling extremely stressed. Stress-induced absenteeism costs German companies about 20 billion Euros annually [5]. Furthermore, not only adults are affected: a comparative study of the WHO found children and adolescents to be frequently tired and exhausted, to have problems falling asleep, and to present as increasingly irritated due to stressful school and life conditions [6].

### 1.1. Stress as a Preparation to Act

Stress is the most widespread disease of the modern age. Scientists have analyzed stress from a biological [7], psychological [8,9] and sociological [10] point of view and have created a number of explanatory theories and models. The most well-known is the transactional model of stress and coping of Richard Lazarus and his research group (e.g., [11,12]). Lazarus and his colleagues conceptualized stress as a consequence of the individual’s appraisal of his or her environment. It emerges, when the individual evaluates a situation or an incident as challenging or threatening (primary appraisal) and his or her own coping abilities as insufficient in the light of the situation’s requirements (secondary appraisal) [13]. Stress management, therefore, is an act of cognitive appraisal, which results in a certain coping behavior [12].

Most recent stress theories integrate biomedical, psychosomatic, cognitive-behavioral, and sociological approaches [2]. Building on appraisal theories [12,13], the recent embodiment theory by Peter Payne and Mardi Crane-Godreau [14], assumes that stress emerges when the organism’s preparation to act and cope—the so called *Preparatory Set*—does not resolve the aversive situation. This could occur, for example, because the behavior is disorganized or inappropriate, or simply because the situation exceeds the coping abilities of the organism (compare the Preparatory Set Theory bv Peter Payne: [14]). A Preparatory Set describes the rapid, largely sub-cortical, organismic preparation to respond to the environment. This preparation involves an organization of physical posture and muscle tone, visceral state, affective or motivational state, arousal and orientation of attention, and (subcortical) cognitive expectations [14]. If the aversive situation is not resolved, the complex activation of the organism is maintained and the organism stays in constant excitation and eventually is burned-out [14]. Because a Preparatory Set involves the entire organism, leverage points to tackle stress and foster resources can be manifold and are not restricted to cognitive-behavioral aspects.

### 1.2. Creative Arts Therapies and Arts Interventions for Stress Management and Prevention

Due to the increasing mobility, flexibility, and performance demands in today’s society, it is assumed that individuals’ stress exposures will increase further in the future [3]. Therefore, innovative and embodied interventions for stress prevention and health promotion are needed more than ever. 

This is where Creative Arts Therapies (CATs) and arts interventions come in as they take into account what Lazarus and colleagues have missed. By conceptualizing body, mind, action, and perception as a unity, CATs, such as Music, Dance/Movement, Art, and Drama Therapy, as well as simple arts interventions, use artistic media to approach the client on a creative and nonverbal level [15]. In addition to cognitive ways of coping, CATs target (en-)active creation, interoception (body experience), and expression in order to access emotions and change behavior (embodied appraisal) [16]. Utilizing a solutogenic approach of health and disease [17], CATs provide action opportunities geared toward health maintenance and focus on health promoting aspects. The recently developed model of embodied aesthetics by Koch [15,18,19] underlines the embodied enactive nature of CATS. Different from conventional therapies, all CATs encourage and enable their clients to actively create or generate. Attention and concentration, for example, are influenced by the perception, exploration, and creation of artistic content as well as by the explicit use of the body (body perception and expression). The respective art media (art, music, dance, theater) thereby provide different methods to activate resources and coping abilities and increase action flexibility, self-efficacy, and empowerment [18,20,21]. See Box 1 for a short overview on the four main CATs and a few exemplary fields of application.

Box 1 **Overview of the four main modalities of CATs.**

**Creative Arts Therapies (CATs)** are generally defined as “the creative use of the artistic media (art, music, drama and dance/movement) as vehicles for non-verbal and/or symbolic communication, within a holding environment, encouraged by a well-defined client-therapist relationship, in order to achieve personal and/or social therapeutic goals appropriate for the individual” ([22], p. 46). CATs can and should be differentiated from arts interventions or artistic activities applied within the context of psychotherapy, counseling, rehabilitation, or medicine [22,23,24]. While arts interventions use the arts to offer primarily artistic experiences with a therapeutic potential, CATs intentionally use the arts to offer therapeutic change. Creative Arts Therapists are registered with an arts therapies professional association and practice within a specific and regulated code of ethics [22]. CATs and arts interventions encourage clients to express themselves creatively, which is why they are also called expressive therapies or expressive interventions [23,25]. For a further clarification of the terms see Karkou and Sanderson’s discussion and illustration of differences and overalppings of CATs and arts interventions ([22], p. 45).

**Music Therapy (MT)** is the targeted application of music (music perception, production, and reproduction) within a therapeutic relationship in order to regain, maintain, and promote physical and psychological health [26]. With the help of different instruments or one’s own voice, emotions and fantasies are expressed and experiences of contact created [26,27]. Music, in this context, furthers the ability to experience oneself and others, symbolize and relate. Clinically MT is, for example, used with psychiatric patients, neurological patients, or with children with autism [28]. In **Art Therapy (AT)**, clients work with different materials (e.g., water colors, crayons, clay). Through the process of creation as well as through relating to one’s own art work, possibilities of expression are created. Previous experiences can be symbolized and expressed in a safe way. Through the fostering of symbolization and (nonverbal) communication, new perspectives and insights can be gained [20,29]. Specifically, the revival and/or fostering of creative resources increase self-efficacy and coping abilities in stressful situations [29]. Clinically, AT is used with patients who have had traumatic experiences or oncological patients among others [30]. **Dance/Movement Therapy (DMT**) is defined as the therapeutic use of movement to further the emotional, cognitive, physical, spiritual and social integration of the individual [31]. Physically, DMT stimulates the vestibular and cardiovascular system [32]. Psychologically, embodied impression and expression improve interoception, body schema, and body image [32]. Exploring one’s own movement abilities and limitations helps to increase emotion regulation, impulse control, and relation to reality (for example for people with schizophrenia, see [33]). Experiencing the own body in aesthetic movement and flow can increase self-efficacy. This is of particular importance for patients with Parkinson’s disease [34].

**Drama Therapy (DT)** also uses the body as a medium. Voice, language, facial expressions and gestures create an “as if”-reality that enables the expression and reflection of old and recent emotions, test-acting, distancing, balancing of different roles, and the expansion of action opportunities [35,36]. Traditionally AT, MT, DT, and DMT are mostly employed in psychiatric and psychosomatic settings. In some countries, such as the UK and Israel, CATs are also widely employed in the school system.

### 1.3. Researching CATs and Arts Interventions in the Context of Stress Prevention and Stress Management

From an empirical point of view, research on CATs and health-restoring arts interventions is metaphorically speaking in its infancy or adolescence. Drawing from experiential knowledge of individual practitioners a grand literature base of case studies and clinical recommendations has developed, with an only recently growing number of evidence-based studies that provide generalizable, and transferable data [37,38]. CATs are used world-wide in a variety of contexts with many different populations. In addition to creative arts therapists, there are artists offering creative interventions to the patients in health institutions. They most often work without a therapeutic professional background, but also use the potential of the arts in order to foster health. In addition, psychologists, physicians, and other health care professions are increasingly discovering the contribution of the arts in their settings and conduct studies to investigate the workings of the “arts in health”. To do justice to the heterogeneity of creative arts intervention studies in health care, this review includes studies on all CATs as well as arts (art, music, dance and drama) interventions. Mere arts interventions include interventions conducted by, for example, an artist (no licensed creative arts therapist) as well as single session interventions that not necessarily have a therapeutic intention. Both, CATs and arts intervention studies are referred to collectively as *creative arts interventions*. To understand creative arts interventions, and to strengthen their development for instance by identifying indications and contraindications, it is indicated to inspect them in various applied contexts. The systematic review at hand provides an overview of evidence-based studies on *stress management* and *stress prevention* through creative arts interventions. Its goal is to promote a dialogue with various health practitioners and institutions on the potential and limitations of creative arts interventions in the context of stress prevention. To the knowledge of the authors this review is the first on the topic.

## 2. Methods

**Figure 1:**
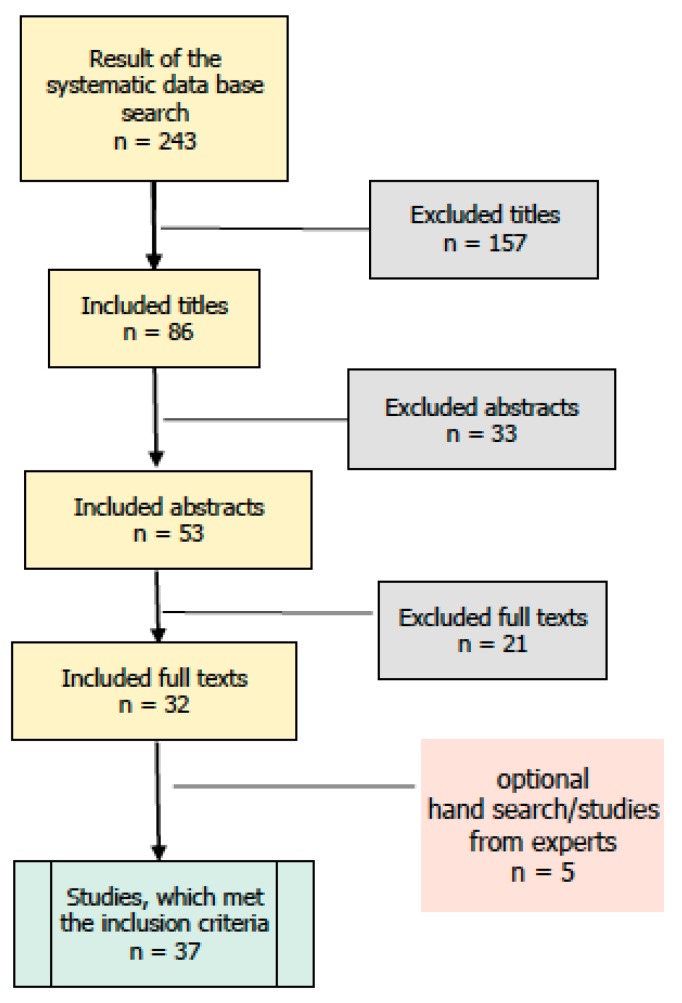
Flow-Chart of the data base search on creative arts interventions for stress prevention.

In a systematic data base search, we collected empirical studies from 1980–2016, which investigated CATs or arts interventions in the context of stress prevention. The cutoff date for the search was the third of August 2016. In addition, we contacted experts in the field and asked them to hand in studies until the end of 2016. The following data bases were searched: Academic Search Complete, ERIC, Medline, Psyndex, PsycINFO, and SocINDEX. Search terms were: art therapy OR art psychotherapy OR creative arts therapies OR drama therapy OR dance therapy OR music therapy AND stress; art AND music AND movement therapy; arts AND health OR mental health treatment OR prevention; arts AND professional education OR training. See Figure 1 for an overview of the search and study selection process. Only studies published in peer-reviewed journals were included.

### Process of the Systematic Study Search

The systematic data base search yielded 243 studies. Studies were scanned following the PICOS principle (patient, intervention, control, outcome, study design) [39]. In a first step, the criterion “patient” was analyzed. To stay in the context of prevention and avoid an overlap with creative arts interventions for acute disorders, for example, in the area of mental health, only studies targeting healthy individuals or people at risk were included. Studies were excluded, if they did not analyze arts interventions in a preventive context. We included both adults and minors, because both groups have been found to be affected by stress. After the first round of exclusions on the base of these criteria, 86 studies remained. In a second step, the studies were scanned according to the criterion “intervention”. Aside from studies that specifically applied CATs, we also included studies, which provided arts interventions (art, dance, music or drama with a group of participants, see reasoning for this decision above). 

In Table 1 CATs are demarcated from mere arts interventions by color coding: clinical studies of CATs are colored green, single session studies of CATs and studies on arts interventions are colored black. Single-session interventions conducted by CATs or other professions were rated as mere arts interventions, because those studies do not fulfill the criterion of containing a therapeutic process with a therapeutic relationship. Interventions neither had to be standardized, nor had to have the same duration. This heterogeneity reflects the variability of creative arts interventions in practice. Fifty-three studies remained. We included studies of evidence levels Ia—III. Evidence levels were defined according to the Agency for Healthcare Research and Quality (AHRQ) [40], rated by the first author and confirmed by the fourth author. Case studies were excluded. Concerning the criteria “control”, “outcome”, and “study-design”, studies should at least operate with a quasi-experimental design, preferably have a control group, and should clearly state their outcome variables. Qualitative, quantitative, as well as mixed method studies were included, as long as their method was clearly stated (in the qualitative realm, e.g., content analysis after Mayring [41]). After checking the remaining studies for those criteria, 32 studies remained. Five studies were handed in by experts after the cut-off date and thoroughly checked for the criteria named above. In total, we analyzed and compared 37 studies.

## 3. Results

Table 1 summarizes content, sample, and intervention characteristics, design, research methods, and results of the studies identified [42,43,44,45,46,47,48,49,50,51,52,53,54,55,56,57,58,59,60,61,62,63,64,65,66,67,68,69,70,71,72,73,74,75,76,77,78]. In total, 37 studies met our inclusion criteria, 11 studies (29.7%) investigated the effect of AT or art interventions on stress, 20 studies (54.1%) focused on MT or musical interventions, and 6 studies (16.2%) assessed the effects of DMT or dance interventions. There were no studies on DT or drama interventions that met the inclusion criteria. In total, 2136 subjects were included: 465 took part in AT or art interventions for stress management, 1241 in MT or musical interventions, and 430 in DMT or dance interventions. Most participants were women (see Table 1). Some studies did not display demographic data of their samples, and were omitted in the respective calculations. The mean age of participants was M = 32.18 years (AT: M = 27.54 years, MT: M = 35.42 years, DMT: M = 33.59 years). Duration times of interventions varied from single sessions on one day to weekly sessions for 10 weeks. Due to the great heterogeneity of interventions and the lack of reported effect sizes in many of the single studies, we did not calculate an overall effect size for the arts modalities. Due to space limitations only the most central qualitative and quantitative results of the included studies are displayed. 

Table 2 gives an analytical overview of the numbers of respective studies by arts modality. As presumed earlier, most of the studies were conducted and published after 2000. Only five studies (13.5%) were published between 1980 and 1999, four of them on musical interventions, one on MT. No meta-analysis (evidence level Ia) was found on CATs or arts interventions for stress management and stress prevention. In total, we found 27 randomized controlled trials (RCTs, evidence level Ib), accounting for 73% of the included studies. Ten of the RCTs analyzed CATs, the remaining seventeen examined the effects of arts interventions. Five studies (13.5%) were rated evidence level IIa (two on CATs, three on arts interventions), two studies (5.4%) were rated evidence level IIb (all on arts interventions), and three studies (8.1%) evidence level III (all on arts interventions). Most of the studies (16; 59.3%) on the highest evidence level came from the area of Music: 9 studies analyzed MT, seven examined music interventions. With eight studies, AT provided 29.6% of the studies on evidence level Ib (none on AT, all on art interventions), and DMT contributed 11.1% (3 studies; one on DMT, two on dance interventions). Within the arts modalities 72.7% (AT and art interventions), 80% (MT and music interventions) and 50% (DMT and dance interventions) of the included studies were RCTs. Most therapy intervention studies (with several intervention sessions) were found in MT (11 out of 20) and one in DMT (1 out of 6). In AT, only studies on effects of artistic activities or single-session interventions were found.

Active **art interventions**, such as drawing or working with clay significantly reduced stress and anxiety in eight out of eleven studies [42,44,45,46,49,50,51,52]. None of the studies analyzed the effects of continuous interventions specifically defined as AT, Three studies [46,51,52] reported significant positive mood changes. Two studies [47,48] did not find a significant stress reduction or mood changes. Two studies [43,51] stated that stress reduction depended on the content of the art work: positive content induced stress reduction, negative content did not. Stress was assessed with various different instruments, such as the Stress Adjective Checklist, the State-Trait Anxiety Inventory, The Global Measure of Perceived Stress, or the Perceived Stress Questionnaire. Two of the studies [45,50] used physical measures (cortisol level [45]; pulse and blood pressure [50]) to operationalize stress. **MT** or **musical interventions** reduced stress and anxiety in 16 of 20 studies (10 on MT, 6 on music interventions) [53,54,56,57,58,59,60,61,62,63,65,66,67,68,70,72]. Four studies (one of MT, three on music interventions) [53,61,68,70] reported reductions in cortisol level, as a physical measurement of stress, and two studies (both on MT) [53,58] found a decrease of sleeping problems through musical interventions. Four studies (one on MT, three on music interventions) [55,59,64] did not find a significant reduction of their stress outcomes.

All studies analyzing **DMT** or **dance interventions** found a significant reduction of stress signs or stress coping abilities in their subjects. Only one of the studies analyzed the effects of DT. In all but two studies [75,76] stress was measured by different self-evaluation tests. In the two other studies, stress was tested with saliva samples for cortisol levels. Four studies [73,74,75,76] furthermore reported a decrease in anxiety levels and negative affect. 

In total, stress was significantly reduced in 30 out of 37 included studies (81.1%). Eleven out of twelve (91.7%) included studies on CATs found a significant stress reduction. Nineteen out of 25 (76%) included studies on mere arts interventions found a significant reduction in their stress measurement. 

## 4. Discussion

Recently, besides being an important part in clinical health care practice, creative arts interventions have become an important area of integrative medicine research. Despite the novelty of CATs, a notable evidence-base on the efficacy of creative arts interventions in various contexts and with many different populations is emerging. 

In the context of stress prevention, the quality of efficacy studies analyzing creative arts interventions is high. Three quarters of the included studies could be allocated to evidence level I and over 80% found a significant improvement in one of their stress-related outcomes. Looking at CATs, conducted by licensed therapists, the percentage rises to more than 90%. Similar to other health care contexts, MT and music interventions contribute the highest quality studies. This can be attributed to the comparably early establishment of MT, its specific focus on group therapy as well as its comparably low psychoanalytic and high empirical orientation (see [25]). CATs and creative arts interventions seem to have a positive impact on perceived stress and stress management. They reduce anxiety levels and improve subjects’ mood. This may be due to certain therapeutic mechanisms that researchers assume to be relevant for all creative arts therapies. Hedonism/play, aesthetic experience/authenticity, nonverbal communication/symbolizing, test-acting in an enactive transitional space, and creation/generativity are named as therapeutic mechanisms, which are active across all CATs ([18,19]; compare [25]). It remains mostly unclear how these therapeutic mechanisms interact or whether they are active in all clients and contexts. Their empirical validation is a task for future research. 

Creative arts interventions’ impact on perceived stress and stress management could not be evaluated by means of an overall effect size for each arts modality. Very few of the studies clearly reported all necessary statistics needed for the calculation of effect sizes. Some did not even report the full demographic data. Furthermore, varying interventions in content and duration impede a final statement on creative arts interventions’ efficacy in the context of stress prevention. This high heterogeneity might be the biggest problem of the young academic field. Being a benefit of the creative approach in practice, the variety of procedures, methods, and interventions makes it hard to assess and judge creative arts interventions’ efficacy with conventional evidence-based research. The high heterogeneity of interventions and measures makes the application of meta-analyses difficult. This is true not only across creative arts interventions but also within each single arts modality (art, music, dance, drama). It might thus be worth looking at specific and common features of the individual CATs, delineating them from mere arts interventions and starting to relate them to features of populations and contexts. This may be a good way to find out, what is specific about each arts modality, and which contexts they work best in (indications and contraindications; see for example [25,78,79,80]). Demarcating core characteristics and mechanisms of CATs or arts interventions individually also helps to choose an adequate control group for intervention studies. Finally, finding commonalities across creative arts interventions could help clarify the benefit they bring to the health system and its agents. Patients already acknowledge these benefits, and the evidence-base on creative arts interventions is in the process of being built.

## Figures and Tables

**Table 1:** Overview of Efficacy Studies on Stress Prevention and Stress Management with Creative Arts Interventions.

Study (Author/Year)	Level of Evidence	Object of Investigation	N/Sample	Design	Intervention	Methods		Exemplary Results
						Data Collection	Analysis	
**ART THERAPY**								
Abbott et al. (2013) [42]	Ib	Effect of an artistic action vs. art reception on the stress level	52 Students, 34f/18m, M = 22.7 years	2 × 2 factorial, randomized, controlled	Active & passive artistic condition: painting/drawing vs. viewing art; active/passive non-artistic condition: puzzle vs. viewing the puzzle pictures; 1 single interven-tion	Stress: Mental Arithmetic Task; Stress Induction Task, Stress Adjective Checklist; Creative Personality Scale; Subjective Stress Scale	Multifactorial Analysis of Covariance (MANCOVA)	Stress reduction sig. higher in the group allocated to active artistic condition. F(2, 44) = 3.45, *p* < 0.05
Curl (2008) [43]	Ib	Short-term stress reduction by focusing on a positive, stress-free experience versus a negative, stressful experience during artistic activity	40 Psychology Students, 30f/10m, M = 19.65 years	2 × 2 factorial, randomized, controlled	4 experimental groups: drawing with positive and negative focus; creating collages with positive and negative focus; 1 single intervention	Stress, anxiety: State Trait Anxiety Inventory; collection of heartrate pre-post Level of focusing on positive and negative experiences: Manipulation Check	*t*-test for independent samples; ANOVA	Focusing on positive experiences while artistic activity leads to stress reduction, regardless of the type of artistic technique. F(1) = 13.76, *p* < 0.01
Huss & Sarid (2014) [44]	Ib	Effect of changing compositional elements (shape, size, color, texture) of a self-designed image of a memory vs. a simple memory (guided imagery) on the experience of stress.	35 Healthcare professionals (doctors, nurses, social workers)	Intervention-evaluation-study, randomized, controlled	Group I (Art Therapy): Constructing a stressful work experience on paper, transforming the experience into a stress-free event; Group II (Guided Imagery): introducing and recalling a stressful work experience, 2 days workshop	Definition of artistic elements: Compositional Elements Scale Discomfort, Stress: Subjective Units of Discomfort Scale (SUDS)	Descriptive: frequency analysis of compositional elements, paired significance tests, mean and standard deviation calculation of individual values	Reduction of stress level in Group I and Group II. The compositional elements of shape, size and color are of particular importance. *X^2^* = 8.61(df = 1), *p* = 0.03; *X^2^* = 7.56(df = 1), *p* = 0.04; *t*-test = 3.27(df = 1), *p* = 0.03, *t*-test = 2.03(df = 1), *p* = 0.04, respectively
Kaimal, Ray & Muniz (2016) [45]	IIb	The impact of visual art making on the cortisol level	39 Students, staff & faculty from a large university, 33f/6m, M = 38.88	Single arm, pre-post-design, quasi-experimental	1 single session: 45 min of art making using collage materials, modeling clay, and/or markers, 15 min for consent and data collection before and after the session	Cortisol Level: Saliva Sample (pre/post; ELISA kit method) Prior Art making experience: limited, some, extensive experience self-reported perceived impact of art making: Written responses from participants (qualitative data) converted into numeric data, entered into quantitative database	*t*-test for paired samples, one-way ANOVA, *t*-test for independent samples, Correlation Analysis:	Sig. reductions in cortisol following the intervention: cortisol levels pre- and posttest differed significantly t(38) = 4.54, *p* < 0.01 No sig. differences based on prior experiences on cortisol levels, F(2, 36) = 0.64, *p* = 0.53 No sig. differences based on gender on changes in cortisol, t(37) = 0.456, *p* = 0.65 Self-reported themes were not strongly correlated with changes in cortisol levels
Kimport & Robbins (2012) [46]	Ib	Effect of a guided intervention with clay on the mood	102 Students of an American University, 74f/28m, M = 22.3 years	2 × 2 × 3 factorial, randomized, controlled	Creation of negative mood by showing a short film; 4 different 5-min interventions: Group A: clay + instruction; Group B: clay without instruction; Group C: stress ball + Instruction; Group D: stress ball without instruction; 1 single session	Mood State, Anxiety: Profile of Mood States (POMS), State-Trait Anxiety Inventory (STAI)	Multifactorial Analysis of Variance (ANOVA)	Editing tone (with and without instruction) reduces negative mood (mood and anxiety) more than working with stress balls; guided interventions (clay and balls) reduce negative mood more than interventions without guidance; POMS: F(1, 98) = 7.6, *p* < 0.05; STAI-S: F(1, 98) = 4.4, *p* < 0.05.
Mercer et al. (2010) [47]	III	Stress reduction through visualization (Visual Journaling)	10 5 medical Students, 5 Lecturers	Single-arm, pre-post-design with follow-up	Visualization of a stress-inducing vs. a stress-free emotion, drawing of these emotions, self-explanatory questions for a better understanding of the stress situation; 1 single intervention	Mood State, Anxiety: quantitative: State-Trait Anxiety Inventory (STAI-Y), the Positive and Negative Affect Schedule (PANAS) qualitative: Questions on stress situation	paired *t*-tests	Non-significant reduction of anxiety and improvement of mood.
Pizarro (2004) [48]	Ib	Comparison of art and writing therapy concerning their effects on psychological and health conditions	45 Students, 27f/18m, M = 19 years	randomized, controlled, pre-post-design with follow-up	2 experimental groups: Induction of a stressful situation based on a text; painting this situation (art-stress) vs. writing about this situation (write-stress); control group: painting a still life after a neutral text, 2 sessions on 2 days	Health, Stress, Physical Symptoms, Mood: General Health Questionaire (GHQ-28), The Global Measure of Perceived Stress (GMPS), Physical Symptoms Inventory (PSI), Profile of Mood States (POMS) (short version) qualitative: Questioning on satisfaction; Questionnaire for the Assessment of stress reduction in art and writing intervention (follow-up)	Analysis of Covariance (ANCOVA)	Sig. reduction of social dysfunction in the writing-stress group: F(2, 37) = 3.17, *p* = 0.05; No health improvements in the art groups: F(2, 37) = 0.10, ns; But more joy and commitment of study participation in the art groups.
Sandmire et al. (2012) [49]	Ib	Reduction of anxiety through artistic activity	57 Students, 45f/12m, M = 18.8 years	randomized, controlled	experimental group: Choice of one of five artistic activities (mandala, free painting, collage, clay, drawing), 1 single session, duration: 30 min.; control group: no intervention	Anxiety: State Trait Anxiety Inventory (STAI)	*t*-tests for paired samples; multifactorial Analysis of Variance (ANOVA)	Sig. reduction of anxiety (state and trait) before final exams. *p* < 0.001, F(1) = 12.72
Schrade et al. (2011) [50]	Ib	Relaxation and reduction of stress levels through painting mandalas.	15 Adults with mental disabilities 9f/10m	Repeated measure, randomized, controlled	3 conditions: painting mandalas, free painting, neutral control condition (puzzle, board games, etc.), 1 single session	Stress: blood pressure, pulse (Sphygmomanometers)	three separate ANOVAs for repeated measures	Sig. reduction of blood pressure (systolic and diastolic) in the mandala group over time: F(2, 28) = 6.05, *p* < 0.05; no sig. change of stress levels comparing the three groups.
Smolarski et al. (2015) [51]	Ib	Effect of instructions on emotional expression on the mood-enhancing qualities of drawing	45 Students, 28f/17m	randomized, controlled, doubleblind, pre-post design	1. Inducing a negative mood 2. Randomized allocation to three groups: Group A: drawing happiness (acting out a positive mood) Group B: drawing current stress (acting out a negative mood) Group C: tracing and coloring a simple drawing (control group, distraction strategy), 1 single session	Mood State: Profile of Mood States (POMS)	two-factorial ANOVA with repeated measures (3 groups; time: baseline, pre-post-treatment)	Sig. mood improvement through the expression of a positive emotion while drawing in comparison to the expression of a negative emotion (venting) or the drawing of simple lines (control group), F(2, 42) = 4.0, *p* < 0.05.
Walsh et al. (2004) [52]	IIb	Effect of an artistic intervention on anxiety and stress among family members of cancer patients	40 family members of cancer patients, 30f/10m, M = 51.43 years	pre-post-design, quasi-experimental	Creative-artistic intervention, e.g., painting mandalas, painting; Implementation in the hospital room of the patient, 1 single intervention	Mood State: Mini-POMS Anxiety: Beck Anxiety Inventory (BAI) Negative and positive emotions: Derogatis Affects Balance Scale (DABS)	*t*-tests for paired samples	Sig. reduction of stress, anxiety: t(40) = −3.42, *p* = 0.001; increase of positive emotions, t(46) = 11.87, *p* < 0.001.
**MUSIC THERAPY**								
Beck, Hansen & Gold (2015) [53]	Ib	Effect of MT on biopsychosocial parameter	20 Danish workers with stress-related incapacity to work, 16f/4m, M = 45.5 years	randomized, controlled	Music Therapeutic Intervention: Guided Imagery and Music (GIM) 2-hour-sessions, 6 sessions in 9 weeks	Cortisol, testosterone, melatonin: analysis of saliva in the laboratory Stress: Perceived Stress Scale (PSS); Profile of Moods States (POMS-37); Visual analogue scale for immediate stress sensation before and after the sessions; Karolinska Sleep Diary (KSQ); Unpublished 16-item scale for physical stress symptoms Willingness to work: single item Well-being: WHO-5 Well-Being Index Anxiety: Generalized Anxiety Disorder 7 (GAD-7) Depression: Major Depression Inventory	*t*-test for independent samples	Sig. improvement in well-being and sig. reduction in sleep disturbance and physical distress. Early intervention leads to faster re-entry of work & positive sig. effects on stress, mood, sleep disorder, depression, anxiety and physical symptoms of distress. (see Table 2, p. 339, in original study)
Brodsky & Sloboda (1997) [54]	Ib	Comparison of effect of Music Therapy and traditional verbal Psychotherapy	55 Musician of a symphony orchestra, M = 36 years	randomized, controlled, pre/post design with follow-up	Three groups: (a) Somatron: traditional verbal psychotherapy, abbreviated progressive relaxation training (APRT), recorded music complemented by music-generated vibrations (b) Music: verbal psychotherapeutic counseling, APRT relaxation exercises supplemented with recorded music (c) Counseling: verbal psychotherapeutic counseling 1 h per week, 8 weeks	Baseline (Anxiety, Stress, Mood State, Burnout): General Health Questionnaire (GHQ-28), Spielberger State Trait Anxiety Inventory (STAI), Derogatis Stress Profile (DSP), Profile of Mood States (POMS), Maslach Burnout Inventory of Music Performer’s Stress (AMPS), and the Music Performance Stress Survey (MPSS) Pre-Post (Mood State, Relaxation): POMS, relaxation exercises	Multifactorial ANOVA with repeated measures	Music-supported forms of therapy as efficient as traditional counseling. 14 of the 52 sets of variables were statistically and clinically significant at measuring time 2 and 3. Differences between groups were not sig. F(2.46) = 4.16; *p* = 0.22.
Brooks, Bradt, Eyre, Hunt & Dileo (2010) [55]	Ib	Effect of MT on self-assessment of burnout, sense of coherence and job satisfaction	65 Medical nursing staff, 43f/9m M = 42.16 years	Randomized, controlled	Guided Imagery with music and relaxation exercises, 3-6 weeks, 60 min sessions	Burnout: Maslach Burnout Inventory Sense of Coherence: Sense of Coherence Scale Job Satisfaction: Job Satisfaction Survey Individual perception of interventions: Self-report on interventions (qualitative survey)	Independent *t*-test Grounded theory	Quantitative results: No sig. differences between experimental group (MT) and control group (waiting). Qualitative results: Music therapeutic Intervention helped subjects to relax and to recharge energy.
Byrnes (1996) [56]	Ib	Effect of audio, video and audio-video stimuli on the stress experience	54 33 Adults (participants of a university summer course) 21 students of music and/or education	Randomized, controlled, pre-post-design	Three groups: (a) Audio-Video: Music-Video excerpt from “Tropical Sweets” (with classical music) (b) Audio: “Aquarium” by Camille Saint-Saens (c) Video: Underwater movie about tropical marine life, 1 single session	Questionnaire on socio-demographic data, music preferences and activities for relaxation, current level of stress Stress Experience Continuous Response Digital Interface (CRDI)	Paired *t*-tests	Stress reduction especially for participants with a high level of stress at the beginning: t = 3.695, df = 53, *p* = 0.001; Sig. reduction in audio-video condition, audio or video condition alone did not sig. affect stress.
Chang, Chen & Huang (2008) [57]	Ib	Effect of MT on the stress level, anxiety and the degree of depression	236 pregnant, Taiwanese women M = 30.48 years	randomized, controlled	EG: passive music therapy intervention: listening to music 2 weeks, 30 min. per day CG: general prenatal treatment without MT	Stress: Perceived Stress Scale (PSS) Anxiety: State Scale of the State-Trait Anxiety Inventory (S-STAI) Depression: Edinburgh Postnatal Depression Scale (EPDS)	Paired *t*-test Analysis of Covariance (ANCOVA)	Stress reduction, anxiety reduction and reduction of the degree of depression sig. higher in the EG with music therapy intervention than in the CG (see Table 4, p. 2585, in the original study).
Du Rousseau et al. (2011) [58]	IIa	Improvement of sleep quality, mood state, everyday functions	41 Law enforcement officers, firefighters, 13f/28m	pre-post-design, controlled	Brain Music (BM) Music-based Neurofeedback Therapy	Insomnia, sleep quality, depression, life satisfaction, everyday functions: Pittsburgh Insomnia Rating Scale, Subjective Sleep Questionnaire, Beck Depression Inventory, Life Satisfaction Scale, Daytime Functioning Scale, 4 weeks intervention	Analysis of Variance (ANOVA), paired *t*-tests	Sig. improvement of sleeping quality, insomnia, mood and everyday functioning (see Table 1, p. 392, in the original study).
Goff et al. (1998) [59]	Ib	Comparison of the effects of music and nitrous oxide on the pain-, anxiety- and stress-levels of subjects during a dental treatment	80 dental patients	randomized, controlled, 2 × 2 factorial	(a) treatment under nitrous oxide and Level 1 = no music/level 2 = with music (b) treatment accompanied by self-selected music (Level 1 = without nitrous oxide, level 2 = with nitrous oxide)	Pain, Anxiety, Stress: Saliva samples before and after treatment for determination of S-IgA concentration (secretory immunoglobulins A)	multifactorial Analysis of Variance (ANOVA)	No sig. differences between the two treatment methods; in women sig. stress reduction with music accompaniment (see Tables 1 and 2, p. 24, in the original study).
Hatta & Nakamura (1991) [60]	Ib	Effect of Anti-stress Music-CDs on stress level	52 Students, 28f/24m	Randomized, controlled, Pre-Post-Design	EG: Classical Music vs. Nature Sounds vs. Pop music; CG: no intervention, single session	Stress, Arousal: Stress/Arousal adjective checklist (SACL)	2-factorial Analysis of Variance (ANOVA)	Sig. reduction of stress and arousal through listening to music, regardless of the type of music, F(9, 144) = 4.25, *p* < 0.01.
Ilie & Rehana (2013) [61]	Ib	Effect of playing music on the iPhone on the acute stress level	54 Students, 27f/27m	Randomized, controlled, 2 × 3 factorial, pre-post-design	Group 1: induction of a stress situation Group 2: no stress induction Each: (a) Pressing the music app “Smule Ocarina” for 10 min, i.e., Playing the melody “Twinkle, Twinkle, Little Star” (b) listening to the melody; (c) Sitting quietly 1 single session	Mood State, Arousal: Profile of Mood States (POMS) Level of Cortisol: Salimetrics Oral Swab (SOS)	Mixed-model ANOVA	Sig. reduction of cortisol level in the stress-induced group by listening to or playing the app compared to the control group, F(1, 65) = 21.54, *p* < 0.001.
Jacobsen, McKinney & Holck (2014) [62]	Ib	Effect of MT on the parent-child interaction and parent-child relationship as well as the stress experience of the parents	18 Parent-child dyads from Denmark with neglected children	Randomized, controlled	EG: music therapeutic Intervention CG: standard treatment without MT 10 weekly sessions, 45 to 50 min.	Parent Competencies: Assessment of Parenting Competencies (APC) Stress experience of parents: Parenting Stress Index (PSI) Parent-Child-Relationship: Parent-Child Relationship Inventory (PCRI)	Multifactorial Analysis of Variance (ANOVA)	Improvement of parent competencies and parent-child interaction, as well as stress reduction in the experimental group with MT intervention higher than in the control group (see pp. 321–326 in original study).
Kim (2008) [63]	Ib	Effect of two music therapy approaches on Music Performance Anxiety (MPA)	30 Music Students (Piano)	Randomized, controlled, pre-post-design	2 Groups (a) improved-music-assisted-desensitization-group (b) music-assisted progressive muscle relaxation (PMR) and imagery-group 6 weekly sessions	Anxiety, Stress, tension, relaxation: Visual Analogue Scale (VAS), State-Trait Anxiety Inventory (STAI), Music Performance Anxiety Questionnaire (MPAQ); Measurement of the finger temperature	Tests of significance, Analysis of Variance (ANOVA)	Sig. reduction of MPA in the music-assisted desensitization group at 6 out of 7 measurement points; Anxiety reduction in the music-assisted PMR group to a lesser extent than in the former group, but sig. for stress and tension level. Level of tension: F = 7.55, *p* = 0.016, df = 1, 14; state anxiety of the STAI, F = 5.57, *p* = 0.033, df = I, 14; finger temperature measure, F = 7.87, *p* = 0.014, df = 1, 14
Lesiuk (2008) [64]	Ib	Effect of listening to self-selected music at the workplace on stress levels	33 air traffic controllers, 2f/31m, M = 34 years	Randomized, controlled; Pre-Post-Design	EG: 15 min. Listening to favorite music, in the break of 4 working shifts in 2 weeks; CG: sitting in silence instead of listening to music	Extraversion, Introversion: Eysenck Personality Inventory; Anxiety: State-Trait-Anxiety Inventory (STAI); Measurement of heart rate, blood pressure, state anxiety and subjectively perceived aviation activity	Multifactorial Analysis of Variance (ANOVA)	No sig. differences between physiological and psychological components in the comparison of both groups; Sig. anxiety reduction (state anxiety) in EG and CG over time, F = 19.22, d.f. = 2, *p* = 0.000, and reduced perception of air traffic in both groups.
Maschi & Bradley (2010) [65]	III	Effect of relaxation drums on well-being, empowerment and social connectedness	31 Social Work Students, 29f/2m	Pre-Post-Design	One single session of 2 h relaxation drumming in the group	Stress, Energy, Empowerment, Bonding: Session Impact Scale	paired *t*-tests	Sig. stress reduction, increased energy, empowerment and a sense of community (see Table 3, p. 61, in the original study).
Mohammadi, Shahabi & Panah (2011) [66]	Ib	Effect of MT on stress, anxiety and the degree of depression	19 residents of a home for elderly, 9f/10m, M = 69.4 years	Randomized, controlled	Experimental Group: MT group intervention (music listening, singing, percussion), 10 weeks of 90 min. daily sessions Control group: standard day-to-day acitivities	Stress, Anxiety & Depression: Depression Anxiety Stress Scale (DASS)	Mann-Whitney U-Test	Stress reduction, anxiety reduction and reduction of the degree of depression sig. higher in the experimental group with MT than in the control group (standard day-to-day activities) (see Table 1, p. 63, in the original study).
Murphy et al. (2014) [67]	Ib	Effect of harp therapy on the stress level and clinical outcome values	181 Women in an in vitro reproduction surgery	Prospective, randomized, controlled, pre-post design	EG: harp therapy for in vitro fertilization CG: standard therapy	Anxiety: State-Trait Anxiety Inventory; Pulse, respiratory rate, blood pressure	*t*-test for paired samples, Wilcoxon rang sum test	Sig. higher reduction of anxiety (state anxiety) over time in the EG than in the CG; Conception rate higher in EG than in CG; positive effect on the acute stress level; no sig. Improvement of heart rate and respiratory rate (see Tables 6–9, pp. 96–97, in the original study).
Rider et al. (1985) [68]	III	Effect of music/progressive muscle relaxation (PMR)/guided imagery (GI) on stress hormones	12 Nurses	Quasi-experimental, pre-post design	20-min program of classical music (audio cassettes) incl. PMR and GI (visualization of imaginative images); 5 times a week over 3 months	Adrenal Corticosteroid (Stress hormones): Urine samples, temperature measurements; Taylor-Johnson Temperament Analysis, State-Trait Anxiety Inventory, Torrance Test of Creativity, Circadian Type Questionnaire	*t*-tests	Reduction of circadian amplitude and corticosteroid temperature rhythms during music listening; The average corticosteroid level did not improve sig. over time.
Sharma & Jagdev (2012) [69]	Ib	Effect of MT on the self-esteem of adolescents	60 adolescents with high school stress levels & low self-esteem, M = 16.85 years	pre-post design, controlled	EG: 30 min listening to classical Indian music (raga, flute) per day, 15 days; CG: discussion of study irrelevant topics	School Stress (Anxiety, Frustration, Pressure Conflict): Scale of Academic Stress (SAS-3) Self Esteem: Self Esteem Inventory (SEI)	*t*-tests	Sig. increase in self-esteem in the EG compared to the CG. (see Table 2, p. 59, in the original study)
Smith & Joyce (2004) [70]	IIa	Effect of MT on the state of relaxation and stress	63 students, 45f/18m, M = 20.88 years	Quasi-experimental, controlled	EG1: receptive MT-Intervention (Mozart). EG2: receptive MT-Intervention (New Age Music) CG: reading offer without MT Relaxation Sessions of 28 min, 3 days in a row, once a day	State of Relaxation and Stress: Smith Relaxation States Inventory (SRSI)	Pearson chi^2^-test	Stress reduction and increase of the relaxation state higher in EG1 (Mozart) compared to EG2 (New Age Music) or to the control group (reading of leisure magazines). (see p. 220)
Smith (2008) [71]	Ib	Effect of MT on Anxiety	80 Employees of a call center 40f/40m, M = 37.5 years	Randomized, controlled	Experimental group: Progressive muscle relaxation with music Control group: verbal discussion, 1 single session	Anxiety: State Trait Anxiety Inventory	*t*-test with repeated measures	Anxiety reduction sig. higher in the EG with musical relaxation intervention than in the CG (verbal discussion): decrease in tense rating: t(39) = 12; *p* < 0.01; increase in pleasant and relaxed rating: t(39) = −20.27; *p* < 0.01; t(39) = −16.2; *p* < 0.01.
Toyoshima, Fukui & Kuda (2011) [72]	Ib	Effect of creative activities on cortisol levels and anxiety	57 Students, 30f/27m, M = 21.5 years	Randomized, controlled	Three experimental groups (1 piano playing, 2 clay modulations, 3 calligraphy) and a control group (lingering in silence) 1 single session	Anxiety: State Trait Anxiety Inventory Detection of cortisol in saliva	Multifactorial Analysis of Variance (ANOVA)	Anxiety reduction and cortisol degradation higher in the EG (creative interventions) than in the CG (lingering in silence). Playing the piano shows the biggest effects. F(1,113) = 5.57, *p* = 0.0202
**DANCE/MOVEMENT THERAPY**								
Bräuninger (2012) [21]	Ib	Improvement of stress management and stress reduction, as well as the influence of DMT group intervention on quality of life (QoL)	N = 162 Clients suffering from stress (self-assessed), 147f/15m M = 44 years	Randomized, controlled, pre-post-design with follow-up after 6 months	EG: DMT, group intervention, 10 weeks CG: waiting control group	Stress management and stress reduction: Stress processing questionnaire/SVF 120 General Stress Level and Psychopathology (Brief Symptom Inventory/BSI) Quality of Life (QoL): The World Health Organization Quality of Life Questionnaire 100 (WHOQOL-100) and Munich Life Dimension List (MLDL)	Stress Data: Multifactorial Analysis of Variance (ANOVA) Quality of Life: Analysis of Variance with repeated measures (repeated measures ANOVA)	Negative stress management strategies decreased sig. in short and long term comparisons, positive strategies of distraction increased, as well as relaxation; sig. short-term improvements in the BSI, especially with regard to anxiety scores; QoL dimensions were sig. better in the EG than in the CG (see Tables 4 and 5, pp. 447–448, in the original study).
Pinninger, Brown & McKinley (2012) [73]	Ib	Comparison of tango and mindfulness meditation regarding stress reduction, reduction of anxiety and depression symptoms and improvement of well-being	Sample N = 79 (self-assessed) depressedPersons, 19.5f/80.5, M = 38.68 years	3 × 2 factorial design, randomized-controlled, pre-post test	Three interventions: Tango: 6 weeks, 1 ½ h per week Mindfulness meditation: 6 weeks, 1 ½ h per week Waiting control group	Anxiety, Depression: Depression, Anxiety and Stress Scale (DASS-21-Scale); Rosenberg Self Esteem Scale; Satisfaction with Life Scale, and Mindful Attention Awareness Scale.	Analysis of Covariance (ANCOVA) and Multiple regression analysis	Significantly reduced depression symptoms in both (F(2, 59) = 6.00, *p* = 0.004), the tango group and the mindfulness group compared to the control group; reduced stress only in the tango group (F(2, 59) = 3.88, *p* = 0.026); participation in the tango dance was a significant predictor of improved mindfulness.
Pinninger, Brown & McKinley (2013) [74]	Ib	Effect of an intensive program of Tango dance on self-reported stress, anxiety or symptoms of depression	N = 41 (experimental group: 20, waiting control group: 21)	Randomized, controlled (RCT) Pre, Post-Test, follow-up after one month	EG: Tango dance program (4 × 1 ½ h in 2 weeks), waiting control group	Self-assessment scales of stress, anxiety and depression symptoms: Depression Anxiety and Stress Scale (DASS-21); Insomnia Severity Index; Satisfaction with Life Scale; General Self-efficacy Scale; Mindful Attention Awareness Scale; Qualitative feedback	Analyzes of Covariance (ANCOVAs)	Self-assessed stress-, anxiety- and depression-symptoms in the experimental group sig. improved compared to the control group; effects were retained at follow-up time (1 month); life satisfaction and self-efficacy sig. improved; mindfulness did not change sig.: stress, F(1,38) = 12.59, *p* = 0.001; anxiety, F(1,38) = 8.31, *p* = 0.006; depression, F(1,38) = 25.60, *p* = 0.001; insomnia, F(1,38) = 8. 30, *p* = 0.006
Quiroga Murcia, Bongard & Kreutz (2009) [75]	IIa	Effects of tango dance on psychophysiological emotion or stress measurements	22 healthy individuals with min. 1 year tango experience, 11f/11m M = 43.09 years	2 × 2 factorial, controlled	4 Conditions: 1. Regular tango dance with partner and music 2. Tango dance with partner without music 3. Dance without partner but with music 4. Movement without a partner and without music 20 min sessions	Stress: Positive and Negative Affect Schedule (PANAS) Saliva samples for the study of cortisol and testosterone	Multivariate Analysis of Variance (MANOVA) with repeated measures	Sig. reduction of negative affect, sig. improvement of positive affect (F(1, 21) = 5.06, *p* < 0.05), and sig. reduction of cortisol concentration in saliva through tango dance with music and partner (F(1, 19)= 5.45, *p* < 0.05). The effect was dependent on the music, but not on the partner.
West et al. (2004) [76]	IIa	Comparison of African Dance and Hatha Yoga regarding their influence on well-being	69 Students 47f/22m M = 19 years	3 × 3 factorial, controlled	3 Conditions: 1. Hatha Yoga 2. African Dance 3. Biology Lecture 90 min courses, single session	Stress: Perceived Stress Scale (PSS), Positive and Negative Affect Schedule (PANAS), saliva samples for the measurement of cortisol	Multivariate Analysis of Variance (MANOVA) with repeated measures	Sig. reduction of perceived stress and negative affect as well as improvement of the positive affect in Hatha Yoga and in African dance: F(2, 66) =11.77, *p* < 0.0001; Sig. reduction in cortisol concentration in saliva in the yoga condition (F(2, 59) = 17.28, *p* < 0.0001); Increase of cortisol in saliva in the dance condition and no change of cortisol in the control condition.
Wiedenhofer & Koch (2016) [77]	IIa	Comparison of non-goal-directed movement and goal-directed movement in terms of their influence on stress and well-being	N = 57 Students, 44f/12m M = 23.21	Two-factorial, controlled, pre-post design	EG: non-goal-directed movement improvisation to music, one-time participation 40–50 min. CG: goal-directed movement improvisation to the same music. Colorful post it-notes were used as target points in the room, one-time participation	Perceived Stress: PSQ30 questionnaire Well-being: HSI (Heidelberg State Inventory) Self-efficacy: General Perceived Self-Efficacy Scale (GSE scale) Body-self-efficacy: Self-Efficacy-Scale (BSE)	MANOVA with repeated measures *t*-tests for paired samples	Perceived stress in EG sig. more reduced than in CG F(56,1) = 4.71, *p* = 0.034 ; Body-Self-efficacy increased sig. in EG F(56,1) = 7.00, *p* = 0.011, no difference in well-being:.

Notes: AT = Art Therapy, MT = Music Therapy, DMT = Dance/Movement Therapy, sig. = significant(ly). Evidence levels are defined according to the Agency for Healthcare Research and Quality (AHRQ) [40]; CATs are demarcated from mere arts interventions by color: clinical studies of CATs are colored green, single session studies of CATs and studies on arts interventions are colored black.

**Table 2:** Overview of the reviewed studies. Evidence levels are defined according to AHRQ [40].

	Time of Publication			Evidence Level			
Arts Modality	1980–1999	2000–2016	Total	Ib	IIa	IIb	III
Art therapy/art interventions	0 (0/0)	11 (0/11)	**11** (0/11)	8 (0/8)	0 (0/0)	2 (0/2)	1 (0/1)
Music therapy/music interventions	5 (1/4)	15 (10/5)	**20** (11/9)	16 (9/7)	2 (2/0)	0 (0/0)	2 (0/2)
Dance/movement therapy/dance interventions	0 (0/0)	6 (1/5)	**6** (1/5)	3 (1/2)	3 (0/3)	0 (0/0)	0 (0/0)
Drama therapy/drama interventions	0 (0/0)	0 (0/0)	**0** (0/0)	0 (0/0)	0 (0/0)	0 (0/0)	0 (0/0)
**Total**	5	32	**37**	27	5	2	3

Note: The bold numbers stand for total numbers
